# Keystone Veterinary Medical Association

**Published:** 1901-03

**Authors:** E. M. Ranck

**Affiliations:** Secretary


Secretary.

### KEYSTONE VETERINARY MEDICAL ASSOCIATION.

THE regular meeting convened at Broad and Filbert Streets, Philadelphia, Pa., January 8, 1901, with the following members of the profession present: Drs. Rhoads, Mahaffy, Harger, Marshall, Hoskins, Carter, Mecray, Underhill, Noack, Eves, Pearson, Adams, Drake, and Ranck.

Dr. C. J. Marshall reported a case of a Jersey heifer which had her first calf when eighteen months old, and at that time had a fair amount of milk. She lately gave birth to her second calf and is giving no milk. Treatment adopted by the doctor is the one suggested by Dr. Pearson in his "Lectures on Practice."

The subject of the recent valuable work of Drs. McCarthy and Ravenel in the rapid diagnosis of rabies by examination of the ganglion of the tenth cranial nerve was discussed, after which a general discussion on rabies ensued, which was entered into by Drs. Eves, Hoskins, Pearson, Noack, Underhill, Drake, Williams, Houldsworth, and others.

Dr. Eves reported the following cases: A mare bitten in November did not develop the symptoms until six months afterward. This case was not proved by subdural inoculations, but showed clear clinical symptoms. About fifty or sixty cows were all bitten by a collie dog, which was afterward killed. One peculiar symptom noticed was that one cow would attempt to bite a stick when thrust at her.

Dr. Pearson said he believes that mad dogs are usually afraid, which accounts largely for the few people bitten, but which also accounts for the large number of animals. One epidemic of rabies was caused by a dog making a circuit of about twelve miles and in one place biting twelve pigs in a pen. The peculiar fact attending this was that they were all bitten about the mouth, due to the fact that pigs always defend themselves by means of their teeth; finally, this dog returned home and was chased away by the children throwing apples at him. Dr. Pearson also thinks that some people who are bitten and do not develop rabies would do so if they did not use precautions in cleansing the wound, and also if some of the saliva were not wiped off on the clothing.

In an outbreak among sheep the following symptoms were noticed: Chewing the wool off the backs of other sheep; very nervous; butting the head against a wall; plunging forward and trying to attack people—one having pinched or bitten his owner's hand, on which he wore a glove, showing an unusual symptom. Infection was caused at night by a dog who did not mangle the sheep, but simply nipped each one on the face.

Dr. Underhill reported a case of rabies in a horse showing the usual symptoms in an aggravated form; the horse was shot. History: A mad dog going through the stables about a month previously.

The question of compensating the owners for dogs or other animals which have rabies and which have to be killed was brought forth by Dr. Pearson, who said the best method so far is to quarantine bitten animals from ninety to one hundred and twenty days. Do not appraise animals showing symptoms. It was suggested to use the dog-tax fund, which should be fixed by the law, to pay for animals bitten, and ordered destroyed. Dr. Mecray, of New Jersey, said that all animals are taxed in that State at the rate of one dollar for dogs and three dollars for open bitches. Further discussion by Drs. Drake, Mahaffy, Noack, and Harger, who closed the discussion.

A case of radial paralysis was reported by Dr. Adams, who said the causes were pressure on the radial nerves from lying down—in heavy horses from pulling; cannot say why this takes place.

History: Standing in the stable for a few days; pulled two or three times hard and stood for about fifteen minutes. Leg collapsed under the weight of the body, but would support the weight when the leg was not flexed—that is, if the knee was well back.

Prognosis: Good in most cases, which usually recover, with some atrophy of the muscles.

Treatment: Stimulating the muscles; two or three grains of veratrine in an aqueous solution, two drachms. This usually produces some irritation and swelling, which disappears, and then inject again.

Dr. Hoskins was called upon for his views of the causes which led to the failure of the bill for the establishing of the "Veterinary Corps" in the U. S. army. He said that the opposition of the Secretary of War had a great deal to do with defeating the bill. The situation in Washington had been most unfortunate, owing to the press taking up the letter from Dr. Morris, of the New York State Veterinary Medical Association, intimating that the society of which he was Secretary was against the bill, which was a falsehood. Secretary Root used this, as a peg on which to hang his hat, in defeating the establishment of an Army Veterinary Corps. Furthermore, Dr. Morris' associates should not bear his actions, and he should be isolated from all Associations. State Associations should take measures which will culminate in his expulsion. There is still hope for veterinarians having rank, but for the establishment of a Veterinary Corps all hope has been abandoned.

Dr. Pearson said he had received a 'phone message from Dr. Salmon, that the bill, as spoken of, is dead, but might get some recognition as lieutenants in the army. He says that the probabilities are that we had been sold out at the last moment by members of the profession like Dr. Morris, who intimated that it was a one-man bill, and this was worked with success in defeating the bill. It seems very unfortunate that one individual should be so situated that he could be so influential. He has been in such peculiar places before, in one instance defeating the bill relative to the control of tuberculosis in New York State by saying that tuberculosis was not infectious, etc. He has no right to speak for an entire State simply because he is Secretary of a State Association; neither has he a right to speak for the entire veterinary profession, simply because by means of his Association he can wield so much power and influence, which acts as a detriment to the profession at large. So a motion, that a committee of three be appointed to draw up a statement expressing the feeling of this Association in regard to the action of the Secretary of the New York State Veterinary Medical Association in the matter of veterinary army legislation was unanimously carried.

The President appointed Drs. Pearson, Harger, and Adams.

A motion was then made to have a Committee on Legislation appointed by President Houldsworth; carried.

Committee appointed to visit the New Jersey Association: Drs. C. J. Marshall, J. M. Carter, and W. Horace Hoskins.

After which the Association adjourned.

*February* 12, 1901. The regular monthly meeting of the Association was held at Broad and Filbert Streets, Philadelphia, on the above date, with Vice-President Marshall in the chair. The following members of the profession were in attendance: Drs. Carter, Hoskins, Mahaffy, Gardner, Marshall, and Ranck. Reading of the minutes was first on the programme, which were approved. There being a Committee on Legislation to be appointed by President Houldsworth, the matter was laid over until some near future date, the Secretary being asked to communicate with Dr. Houldsworth in regard to the same.

The report of the committee which visited the New Jersey Association was next in order. Drs. Marshall and Carter were there, but left while the election of officers was still in progress. Dr. Hoskins, who arrived later, reported about as follows:

First, the election of such an able man as Dr. Lowe as President, and who made an excellent address appealing to the profession at large in New Jersey to wake up and raise the standard and further the interests of agriculturists.

Second, the appointment of a Committee on Animal Industry by President Lowe, the function of which is to attend farmers' meetings, instruct live-stock owners, educate the public to the points relative to infectious and contagious diseases, and act as a general sanitary committee.

Third, the action which this Association plans to take toward making the meeting of the American Veterinary Medical Association in Atlantic City in September a successful one.

Owing to the limited time, Dr. Hoskins was compelled to leave before the meeting adjourned, and so could report nothing further except that the general tenor of the New Jersey Association was to score the action of Dr. Morris, of the New York Association, relative to the Army bill.

A general discussion on tuberculosis brought forth the question by Dr. Hoskins, as follows: "Whether, when examining a herd of cattle for tuberculosis, and finding one or more which on post-mortem prove to have been chronic, we are justified in using a re-test or stronger tuberculin." The consensus of opinion was that we should. He related an experience of recent date in testing a herd of cattle in New Jersey and the herd was destroyed, but probably the party owning the cattle would not be compensated for them. This fact is explained by the inefficient law on this subject in that State, and until something is done by the legislative body the probabilities are that the dread disease will not be under control. Dr. Gardner explained that when a veterinarian discovers any tuberculous cattle the authorities are sent for; they examine them physically only, the worst ones being picked out and killed, for which the State pays something, while the others are left to spread the contagion broadcast. The result of this is that the practitioners, as a rule, do not report the cases, for it does nothing toward stamping out the disease.

Cases reported by Dr. Marshall some time ago, on the operative measures in spavin, including the neurectomies, were again discussed by him. He says the horses are lame again and the operation is probably a failure.

Report, by Dr. Mahaffy, of a case of Cæsarean section in a cow which died later, and also one which has been operated on about seven days ago, but which is living yet.

Adjourned. E. M. RANCK,
Secretary.

